# The complete mitochondrial genome of *Polyozellus multiplex* (Thelephorales)

**DOI:** 10.1080/23802359.2022.2086077

**Published:** 2022-06-30

**Authors:** Jian-Wei Liu, Thatsanee Luangharn, Shi-Mei Yang, Fu-Qiang Yu

**Affiliations:** a Center of Excellence in Fungal Research, Mae Fah Luang University, Chiang Rai, Thailand; b School of Science, Mae Fah Luang University, Chiang Rai, Thailand; c The Germplasm Bank of Wild Species, Yunnan Key Laboratory for Fungal Diversity and Green Development, Kunming Institute of Botany, Chinese Academy of Sciences, Kunming, China

**Keywords:** Mitochondrial genome, phylogenetic relationships, wild edible mushroom

## Abstract

The complete mitogenome of *Polyozellus multiplex* (Underw.) Murrill 1910, was first sequenced, assembled, and annotated in the present study. The mitogenome length was 47,054 bp with a GC content of 23.35%, including 14 conserved protein-coding genes, one ribosomal protein (RPS3), two DNA polymerases (DPO), two rRNA genes (RNS and RNL), and 24 transfer RNA (tRNA) genes. Phylogenetic analysis, based on a combined mitochondrial gene dataset from 17 taxa of four orders within the class Agaricomycetes, was conducted using maximum-likelihood (ML) and Bayesian inference (BI) methods. It is revealed that *P. multiplex* is closely related to *Thelephora aurantiotincta* Corner 1968, both of them have been clustered into Thelephorales.

*Polyozellus multiplex* (Underw.) Murrill. was originally described from Mount Desert, Maine, USA and classified in the genus *Cantharellus* Adans. ex Fr. 1821 with *Cantharellus multiplex* Underw. 1899 (Underwood 1899). Then, Murrill (1910) classified this fungus to a new genus, *Polyozellus* Murrill. 1910, as its cespitose imbricate habit differed from that of other species of *Cantharellus*. Imazeki (1953) reclassified this genus to the *Thelephora* Ehrh. ex Wild. 1787 group according to its lobed nodulose basidiospores thelephoric acid content, which differed from other *Cantharellus* species. Since then, *Polyozellus* has remained a monotypic genus. Voitk et al. (2017) revealed that *P*. *multiplex* was a species complex including five phylogenetic species, viz. *P. multiplex*, *P. atrolazulinus* Trudell & Kõljalg 2018, *P. mariae* Voitk & Kõljalg 2018, *P. marymargaretae* Beug & I. Saar 2018 and *P. purpureoniger* Spirin & I. Saar 2018. Among them, *P*. *multiplex* is a good edible fungus distributing in eastern Asia and northeastern North America (Yang 1992). Here, the mitochondrial genome of *P*. *multiplex* is first reported to promote our understanding in *Polyozellus* taxonomy and genetics.

Two genes (ITS1-5.8S-ITS2: OL913875, LSU rDNA: OL913876) were combined to identify *P. multiplex*, which was from the Dashaba Agriculture Market (104°5′48″E; 26°13′17″N), Xuanwei City, Yunnan, China. In accordance with guidelines and regulations provided by Kunming Institute of Botany, Chinese Academy of Sciences and the local government agency, the fungus collection from this local market was performed without a required ethical approval or other relevant permissions from a national or local agency. The dried specimen (voucher # HKAS122651) was deposited in the Herbarium of Cryptogams, Kunming Institute of Botany, Chinese Academy of Sciences (http://www.kib.ac.cn/, the contact person and e-mail: Mr. Tao Deng, dengtao@mail.kib.ac.cn).

The total genomic DNA of this dried *P. multiplex* specimen was extracted using the CTAB method (Doyle 1987). The whole-genome sequencing was performed on an Illumina sequencing platform (HiSeq PE150) with standard procedures. The 150 bp paired-end libraries were prepared to generate approximately 8 GB of raw data. The mitochondrial genome was assembled by GetOrganelle v. 1.7.5 with the default parameters (Jin et al. 2020). The mitogenome was annotated by the MFannot tool (http://megasun.bch.umontreal.ca/cgi-bin/mfannot/mfannotInterface.pl) and was compared with the result annotated from the reference sequence *Thelephora aurantiotincta* with manual corrections in Geneious Prime 2020.0.3 (BioMatters, Ltd., Auckland, New Zealand).

The complete mitochondrial genome sequence of *Polyozellus multiplex* has been deposited in GenBank (GenBank accession no. OL790394). The circular genome (47,054 bp) comprised of 14 conserved protein-coding genes (PCGs), one ribosomal protein (RPS3), two DNA polymerases (DPO), two rRNA genes (RNS and RNL), and 24 transfer RNA (tRNA) genes. The 14 conserved PCGs respectively encoded the seven ubiquinone reductase subunits of NADH (NAD1, NAD2, NAD3, NAD4, NAD4L, NAD5, and NAD6), three cytochrome oxidase subunits (COX1, COX2, and COX3), three ATP synthase subunits (ATP6, ATP8, and ATP9), and the apocytochrome b (COB). The 24 tRNA genes (tRNA^Ala(UGC)^, tRNA^Cys(GCA)^, tRNA^Asp(GUC)^, tRNA^Glu(UUC)^, tRNA^Phe(GAA)^, tRNA^Gly(UCC)^, tRNA^His(GUG)^, tRNA^Ile(GAU)^, tRNA^Lys(UUU)^, tRNA^Leu(UAA)^, tRNA^Leu(UAG)^, tRNA^Met(CAU)^, tRNA^Asn(GUU)^, tRNA^Pro(UGG)^, tRNA^Gln(UUG)^, tRNA^Arg(UCG)^, tRNA^Arg(UCU)^, tRNA^Ser(GCU)^, tRNA^Ser(UGA)^, tRNA^Thr(UGU)^, tRNA^Val(UAC)^, tRNA^Trp(CCA)^, tRNA^Trp(UCA)^, and tRNA^Tyr(GUA)^) ranged in size from 71 bp to 86 bp, and covered all 20 standard amino acids. The overall base composition is as follows: 37.81% A, 38.84% T, 11.30% C, and 12.05% G, with a GC content of 23.35%. To validate the phylogenetic status of *P*. *multiplex* in Basidiomycota, we constructed a phylogenetic tree for 17 Basidiomycete species. *Sanghuangporus sanghuang* (Sheng H. Wu, T. Hatt. & Y. C. Dai) Sheng H. Wu, L. W. Zhou & Y. C. Dai 2015 and *S*. *vaninii* (Ljub.) L.W. Zhou & Y. C. Dai 2015 were selected as the outgroup. Mitogenomic sequences of *P*. *multiplex* and its allies were extracted, aligned, and concatenated using Geneious Prime 2020.0.3. Maximum-likelihood (ML) and Bayesian inference (BI) analyses of the *P*. *multiplex*’s phylogenetic tree (Figure 1) were performed by using IQ-TREE 1.6 (Trifinopoulos et al. 2016) (http://iqtree.cibiv.univie.ac.at/). A close relationship between *P*. *multiplex* and *Thelephora aurantiotincta* (NC054311) was exhibited in the phylogenetic tree, both of them belonged to Thelephorales.

**Figure 1.**
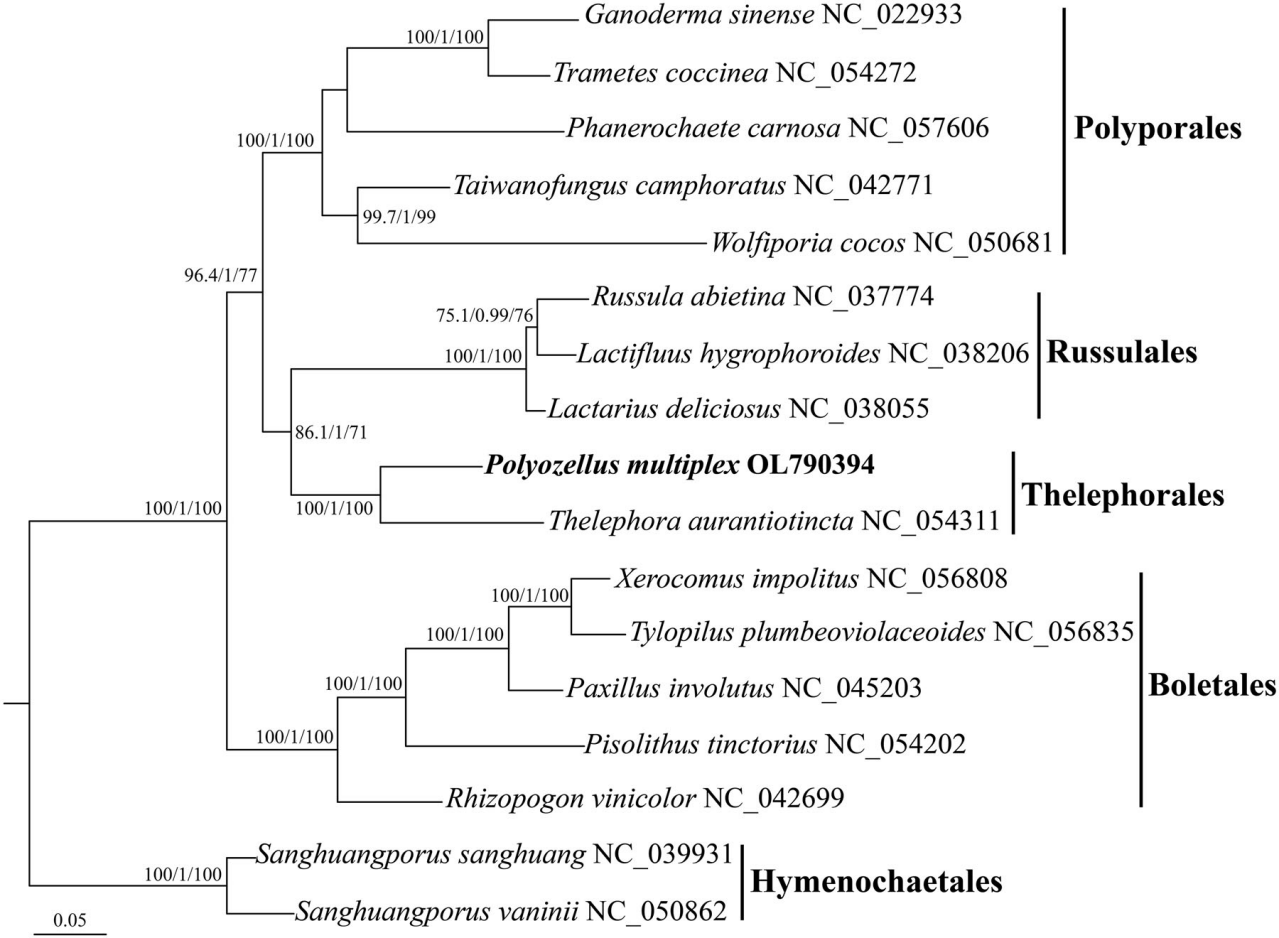
Phylogenetic relationships among 17 species based on concatenated mitochondrial protein-coding genes (PCGs). The 14 PCGs included subunits of the respiratory chain complexes (COB, COX1, COX2, and COX3), ATPase subunits (ATP6, ATP8, and ATP9), NADH: quinone reductase subunits (NAD1, NAD2, NAD3, NAD4, NAD4L, NAD5, and NAD6), with SH-aLRT values (left), PPs values PP (middle), and ultrafast bootstrap values UFB (right) near the corresponding node. Accession numbers of mitochondrial sequences used in the phylogenetic analysis are listed in brackets after species.

## Author contributions

Jian-Wei Liu and Fuqiang Yu designed the study, Jian-Wei Liu and Shi-Mei Yang analyzed the data. Jian-Wei Liu and Thatsanee Luangharn wrote, while Fuqiang Yu revised the manuscript. All authors agree to be accountable for all aspects of the work.

## Data Availability

The genome sequence data that support the findings of this study are openly available in GenBank of NCBI at https://dataview.ncbi.nlm.nih.gov/ under the accession no. OL790394. The associated BioProject, SRA, and Bio-Sample numbers are PRJNA811355, SRS12233056, and SAMN26549901, respectively.
